# NO Activates the Triterpenoid Biosynthetic Pathway in *Inonotus obliquus* Through Multilevel Signaling Regulation to Enhance Its Production

**DOI:** 10.3390/ijms26104561

**Published:** 2025-05-09

**Authors:** Ping Kou, Yu-Chi Zhang, He Wang, Li-Li Mo, Jun-Jiao Gu, Fang Yu

**Affiliations:** School of Biological Engineering, Dalian Polytechnic University, Dalian 116034, China

**Keywords:** *I. obliquus*, NO, triterpenoid, signal pathway

## Abstract

Triterpenoids are the bioactive components in *Inonotus obliquus* with extensive medicinal prospects, but their low content in fermentation production is the main limiting factor for their application. This study focuses on nitric oxide (NO), an important signaling molecule within organisms, aiming to explore its inducing effect on the synthesis of triterpenes in *I. obliquus* and the potential signaling transduction mechanisms involved. Compared with the control group, the content of representative triterpenoid betulin increased by 70.59% after adding the NO donor sodium nitroprusside. Gene expression level detection revealed that NO mainly promotes its biosynthesis by activating the transcription of key enzyme genes in the downstream pathway of betulin biosynthesis, thereby increasing its abundance. Tracing upstream, the NO signal was found to induce the upregulation of genes related to cellular antioxidant and calcium ion signaling pathways. Notably, *IoCAMP* responded strongly to the NO signal, participating in the regulation of cytoplasmic Ca^2+^ concentration by altering the Ca^2+^ concentration of mitochondria together with *IoCATP* and *IoCALM.* Additionally, the signaling of changes in Ca^2+^ concentrations is likely to crosstalk with the reactive oxygen species (ROS) signaling pathway. The increase in enzyme activity of *IoNOX* after NO induction confirmed the activation of the ROS signaling pathway. It works in synergy with *IoSOD* and *IoCAT* to reduce oxidative damage and promote downstream triterpenoid biosynthesis. This study not only contributes to clarify the signaling pathways regulating NO-mediated triterpenoid biosynthesis but also provides a theoretical basis for the efficient production of triterpenoid active components in *I. obliquus*.

## 1. Introduction

*Inonotus obliquus* is a kind of wood-decaying fungus that corrodes birch trees, and it is also an edible and medicinal mushroom [1]. Due to its extensive biological activities, it has a long history of health care and medicinal use in Russia and Eastern Europe [2]. Chemical composition analysis and pharmacological studies have shown that *I. obliquus* can produce a variety of metabolites with biological activity, such as triterpenoids and polysaccharides [3,4,5]. To date, more than 30 triterpenoid compounds, including inotodiol, betulin, and betulinic acid, have been identified in *I. obliquus*, and these compounds have been proven to possess health benefits such as antitumor, antioxidant, immunomodulatory, and hypoglycemic effects [6,7,8]. Therefore, increasing the accumulation of triterpenoid bioactive components in *I. obliquus* is crucial for its development and utilization as functional food.

Due to the need to parasitize live birch trees and slow the growth of fruiting bodies, wild *I. obliquus* lacks sufficient raw materials for extracting active ingredients [9]. To meet the demands of the pharmaceutical and food industries, liquid fermentation is currently the main method for producing specific active triterpenoid components [10,11]. However, the low content of the target components in mycelium remains a bottleneck problem limiting the application of triterpenoids in *I. obliquus* [12]. Therefore, it is crucial to increase the yield of triterpenoids by promoting their biosynthesis.

The biosynthetic pathway of triterpenoids begins with acetyl-CoA. The continuous catalytic pathway for the synthesis of triterpenoids from acetyl-CoA can be divided into two reaction stages (Figure S1). The mevalonate pathway (MVA) from acetyl-CoA to IPP can be classified as the upstream stage, which is relatively far from the downstream triterpenoid biosynthesis. It is catalyzed successively by acetoacetyl-CoA transferase (AACT), hydroxymethylglutaryl-CoA synthase (HMGS), hydroxymethylglutaryl-CoA reductase (HMGR), mevalonate kinase (MVK), phosphomevalonate kinase (PMK), and diphospho mevalonate decarboxylase (MVD), ultimately generating isopentenyl diphosphate (IPP). The synthesis of triterpenoids from IPP can be divided into the downstream stage. In other species such as *Betula platyphylla Suk*, the functions of downstream enzyme genes have been basically fully analyzed. The enzyme genes in this stage include farnesyl diphosphate synthase (FPP), squalene synthase (SQS), and squalene epoxidase (SQE), which produce 2,3-oxidosqualene. Then, different enzymes catalyze the synthesis of triterpenoids with a different parent. Currently, only the lupeol synthase (LUS) has been cloned and identified in *I. obliquus*. Therefore, studying the response mode of triterpenoids with the lupeol structure to environmental signals can better reflect the specific regulation of environmental signals on the triterpenoid synthesis of *I. obliquus*.

The production of these triterpenoid active components, including betulin, may be related to the frequent exposure of *I. obliquus* to environmental stresses such as low temperatures, ultraviolet radiation, and invasion by pathogenic microorganisms, but the specific stress response processes are not yet clear [13,14,15]. Moreover, the biosynthetic pathway of triterpenoids is complex, and subtle differences in the fermentation process may lead to changes in metabolic flux and product accumulation [16,17]. Currently, the main strategies for achieving high yields of triterpenoids in fungi is usually to add inducers in addition to optimizing fermentation conditions [18,19,20]. For example, the addition of methyl jasmonate increased the content of lanostane triterpenoids in *I. obliquus* by 68.50%, and the induction with oleic acid doubled its total triterpenoid content [21]. Although some exogenous abiotic elicitors play an important role in promoting the production of triterpenoids in *I. obliquus*, their regulatory effects and potential mechanisms on triterpenoid biosynthesis are still lacking.

Nitric oxide (NO), as an endogenous small molecule commonly present in organisms, can participate in a series of signal transduction pathways as an important intracellular and intercellular signaling molecule, thereby directly or indirectly regulating various physiological and biochemical processes [22,23,24]. For example, the addition of NO donors can alleviate the oxidative damage caused by high-temperature stress in Pleurotus ostreatus, which is related to the change in the activity of intracellular antioxidant enzymes [25]. Additionally, a few studies have shown that NO can act as an important signal to regulate secondary fungal metabolic processes [26,27]. For instance, the co-cultivation of *I. obliquus* with Trametes versicolor induces NO burst and promotes the synthesis of phenolic compounds [28]. But the role and mechanism of NO in regulating fungal triterpenoid biosynthesis have only been explored in *Ganoderma lucidum*, such as the addition of coix seed oil, which increased the concentration of NO, expression level of CYP450, and total triterpenoid content, and NO was involved in the signal transduction process of coix-seed-oil-induced triterpenoid synthesis in *Ganoderma lucidum* [29]. However, the effect and potential mechanism of NO on triterpenoid biosynthesis in *I. obliquus* are still unclear.

Therefore, the purpose of this study is to investigate the effect of NO donor sodium nitroprusside as an elicitor on triterpenoid synthesis by adding it to the fermentation medium of *I. obliquus*. Betulin was selected as a representative active component to clarify the impact of NO on its accumulation and the expression level of biosynthetic pathway enzyme genes. Furthermore, the upstream potential mechanisms of NO-regulating triterpenoid biosynthetic enzyme genes were explored at the levels of the antioxidant system and calcium ion signal transduction pathways. This study can lay the foundation for the application of inducers that cause endogenous bursts of NO to promote the production of triterpenoid components in *I. obliquus* and provide a basis for exploring the potential regulatory mechanisms of NO-mediated fungal triterpenoid synthesis.

## 2. Results

### 2.1. Effect of NO Induction on the Growth and Terpenoid Active Ingredients Contents of I. obliquus

**Figure 1:**
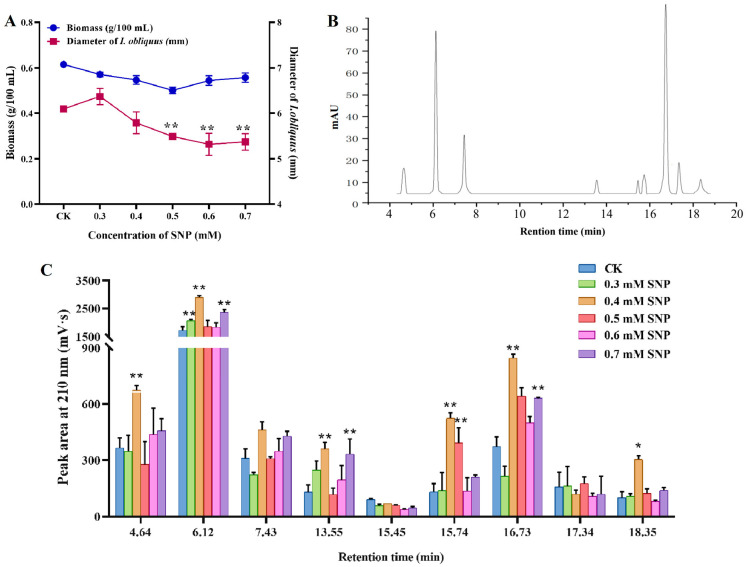
Effects of different concentrations of SNP on the strain biomass and diameter (**A**), high performance liquid chromatography profile at 210 nm (**B**), and content of triterpenoids abundance (**C**). CK indicates the control group check without SNP. ** represents significant differences at the *p* < 0.01 level and * represents significant differences at the *p* < 0.05 level.

There are different growth and metabolism stages in the process of microbial fermentation. Therefore, the time point of logarithmic growth should be determined by the variation of biomass of *I. obliquus* with the extension of culture time (Figure S2). The optimal harvest time of *I. obliquus* was 10 days according to the growth curve, which could be used to investigate the effect of NO induction on the synthesis of bioactive components and the related regulatory mechanism. Figure 1A shows the effects of different concentrations of sodium nitroprusside (SNP) on the strain biomass and mycelium pellet diameter. The biomass and diameter of mycelium decreased with different concentrations of SNP treatment. The diameter of mycelium decreased significantly when the SNP concentration exceeded 0.5 mM, while the difference of biomass before and after induction was not significant.

The nine triterpenoid compounds detected by HPLC were sorted by retention time (Figure 1B), with the peaks corresponding to retention times of 6.12 min, 7.43 min, and 16.73 min showing the highest response values. Figure 1C illustrates the effect of SNP on the intracellular content of triterpenoid active components. As the concentration of SNP increased, the abundance of triterpenoid compounds generally showed an upward trend, but each compound exhibited different patterns of change. Notably, the compound with a retention time of 6.12 min and 16.73 min demonstrated the most significant change in response value after the addition of varying concentrations of SNP. At 0.4 mM, its peak area was the highest, increasing by 68.47% and 127.40% compared to the control group. These results indicate that the addition of SNP effectively promotes the accumulation of triterpenoid compounds, which may be related to the complex regulatory process of triterpenoid metabolism mediated by NO.

### 2.2. NO Induction Enhanced Production of Betulin in I. obliquus

The abundance of triterpenoids in *I. obliquus* was changed by NO induction. In order to further clarify the effect pattern of NO on the triterpenoids of *I. obliquus*, the triterpenoid with the most significant difference in peak area under induction was selected as the representative component to be further analyzed qualitatively and quantitatively. Firstly, the identified compounds in *I. obliquus* were selected as standards (betulin, betulinic acid, inotodiol) for retention time comparison. The compound with a retention time of 16.73 min had the same retention time as betulin and was identified as betulin. However, the compound with a retention time of 6.12 min did not match the candidate standard, and it might have been other unidentified specific active components in *I. obliquus*. Considering the consistency of the biosynthetic pathways of various triterpenoids and the lag in the identification of enzyme genes in *I. obliquus*, finally, the betulin, of which the synthase gene (LUS) has been identified, was used as a representative component for subsequent quantitative analysis and gene expression determination. The quantitative analysis of betulin was performed by the external standard method.

**Figure 2:**
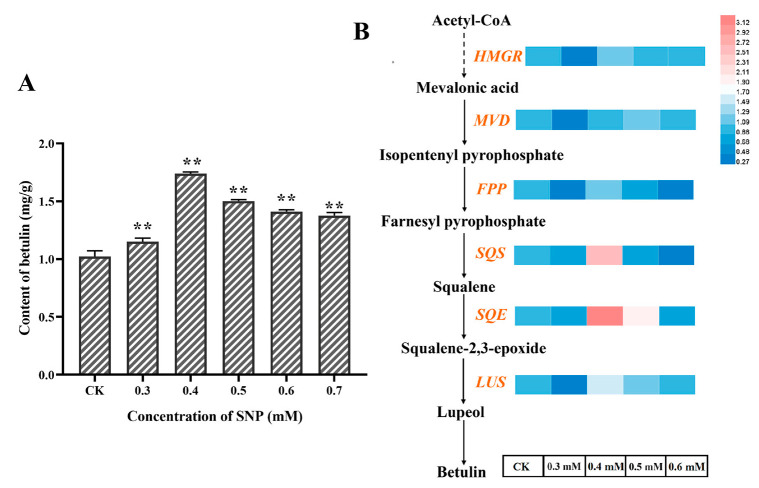
Effects of different concentrations of SNP on the betulin content (**A**) and transcription level of betulin biosynthesis pathway genes after SNP induction (**B**). The normal arrows represents a one-step enzymatic reaction, while a dashed arrow indicates multiple enzymatic reactions. ** represents significant differences at the *p* < 0.01 level.

The effect of SNP at different concentration on the content of betulin in the fermentation process of *I. obliquus* is shown in Figure 2A. The content of betulin increased and then decreased with the increase of SNP concentration. The highest content of betulin was found when SNP was 0.4 mM, which was increased by 70.59% compared with the control group. In order to investigate the induction mechanism of NO on the *I. obliquus* fermentation process more clearly, a high-yield betulin fermentation condition should be established to enhance the induction effect. Figure S3 shows that the content of betulin reached 2.40 mg/g after the optimization of carbon source concentration, nitrogen source concentration, and pH, which was 37.93% higher than before optimization. The established fermentation conditions based on NO induced with a high content of betulin was used as a unified culture condition to further explore the regulatory mechanism of NO promoting the accumulation of betulin in *I. obliquus*.

### 2.3. Transcriptional Response of Betulin Biosynthesis Enzyme Genes Induced by NO

The change of betulin accumulation induced by NO may be closely related to the expression level of key enzyme genes in the biosynthesis pathway. The MVA is the direct pathway for the synthesis of triterpenes. The pathway for the continuous catalysis of acetyl-CoA to synthesize betulin can be divided into two reaction stages. The stage from acetyl-CoA to IPP can be classified as the upstream stage, which is relatively far from the downstream triterpenoid biosynthesis, and thus, only the recognized rate-limiting enzyme genes *HMGR* and *MVD* in this stage were selected for transcriptional level detection. The synthesis from IPP to betulin can be divided into the downstream stage. In other species such as *Betula platyphylla*, the functions of downstream pathway enzyme genes have been basically fully analyzed, but the genomic annotation information and functional identification of the genes in *I. obliquus* are relatively lagging behind. Currently, the known synthase genes in *I. obliquus* in this stage are only *FPP*, *SQS*, *SQE*, and *LUS*. Therefore, the transcriptional expression levels of these four genes were investigated in this study.

Six enzyme genes involved in betulin biosynthesis (*HMGR*, *MVD*, *FPP*, *SQS*, *SQE*, and *LUS*) were selected for qRT-PCR analysis to investigate their transcriptional expression under the induction of different SNP concentrations. Figure 2B shows that the expression of the six related enzyme genes did not change significantly at a SNP concentration of 0.3 mM. This indicates that low concentrations of NO signals cannot induce the upregulation of triterpene biosynthesis enzyme gene expression. At this time, *I. obliquus* may adapt to the environment by adjusting its physiological status or other conventional metabolite syntheses.

The expression level of the target genes was upregulated with the increase of concentration (0.4–0.5 mM). The expression of five downstream enzyme genes, *HMGR*, *FPP*, *SQS*, *SQE*, and *LUS* all reached the highest at 0.4 mM, which was consistent with the accumulation profile of betulin. Among them, *SQS*, *SQE*, and *LUS* showed the most active transcriptional response induced by NO with similar profiles, suggesting that the coordinated upregulation of these three genes may be the basic reason for the significant increase in betulin accumulation under NO induction. The specific process may be that these three genes respond strongly at a concentration of 0.4 mM, thereby enhancing catalytic activity and increasing the metabolic flux of intermediate products (farnesyl pyrophosphate, squalene, squalene-2,3-epoxide, lupeol), and consequently enhancing the accumulation of betulin.

Furthermore, the gene expression level significantly decreased when the SNP concentration was 0.6 mM. This shows that the excessive burst of NO (0.5–0.7 mM) may act as a stressor that primarily modulates the response processes at the level of growth and development, thereby weakening the metabolic regulation of triterpene biosynthesis. It is worth noting that the high concentration of SNP forced *I. obliquus* to respond mainly at the physiological level to ensure vitality, unable to fully mobilize the secondary metabolic process. Therefore, the decrease in the expression of these enzyme genes slowed down the synthesis speed of triterpenoid compounds such as betulin, generally reflected in the slow increase in content. However, the accumulation of betulin in this study remained relatively stable at high SNP concentrations, and this unequal repression phenomenon might be achieved by regulating the metabolic flux to avoid the excessive accumulation of triterpenoids, which could harm *I. obliquus* itself. At the same time, because the presence of cytotoxicity of triterpenoid compounds can improve the defense capability, the betulin and other triterpenoids that have been synthesized in the early stage of stress still accumulate in the *I. obliquus* to cope with environmental stress, which is reflected in the accumulation of betulin maintained at a stable range.

### 2.4. Antioxidant Response of I. obliquus Under NO Induction

**Figure 3:**
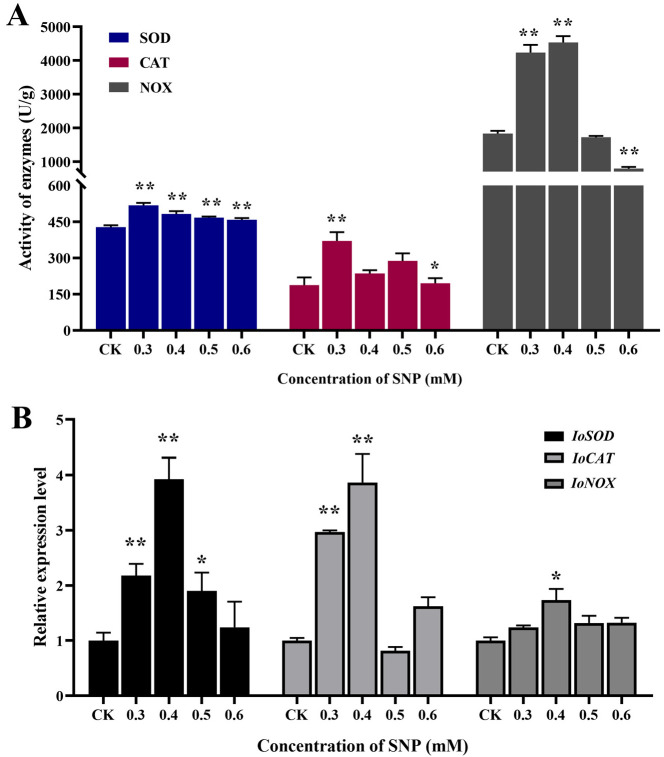
Effects of different concentrations of SNP on the antioxidant enzyme SOD, CAT, and NOX activities (**A**), and transcriptional response of antioxidant enzyme coding genes (**B**). ** represents significant differences at the *p* < 0.01 level and * represents significant differences at the *p* < 0.05 level.

The addition of NO as elicitors can induce the defense response of fungal cells, and the oxidative burst is an important physiological and biochemical reaction in the defense process of fungi. Three antioxidant-related enzymes that can reflect the state of oxidative stress in *I. obliquus* cells were selected for activity assays, and the oxidative defense responses at different SNP concentrations were investigated. Figure 3A shows that the activities of superoxide dismutase (SOD), catalase (CAT), and NADPH oxidase (NOX) were all increased in different degrees under NO induction, especially when the concentration was between 0.3 mM and 0.5 mM. The activity of NOX was higher at 0.3 mM and 0.4 mM, which had a certain concentration delay compared with the other two enzymes. It may be that NOX has a wider response range under NO induction. In particular, the activity of NOX increased by 148% compared with that in the control group, which was significantly higher than that of SOD and CAT (21.22% and 97.60%). This indicates that NOX, as a major enzyme that can produce reactive oxygen species, can respond more strongly to NO induction and produce a large amount of superoxide anions and other reactive oxygen species through redox reactions. The activities of SOD and CAT reached the highest level at 0.3 mM. These results suggest that the antioxidant defense system involving SOD and CAT is more sensitive to NO induction than secondary metabolites, and it can clear excess reactive oxygen species by increasing enzyme activity, thereby reducing cellular oxidative damage at lower SNP concentrations.

The expression levels of the coding genes of these three antioxidant-related enzymes were detected to explore the oxidative defense responses of *I. obliquus* to different SNP concentrations at the gene level. Figure 3B shows that the expression levels of the three antioxidant genes were higher at concentrations of 0.3 mM and 0.4 mM, generally consistent with the response profile of antioxidant enzyme activity. The transcriptional expression levels of *IoSOD* and *IoCAT* were upregulated 3.93-fold and 3.87-fold of the control group at 0.4 mM, respectively. This indicates that the response of SOD and CAT to NO induction at the time of sampling mainly occurred at the gene transcription stage, and the response was strong, laying the foundation for further expression to produce a large amount of antioxidant enzymes. The expression level of the *IoNOX* gene was only upregulated by 73%, but its enzyme activity was the strongest. This indicated that *IoNOX* was the most sensitive and rapid in response, and already produced a large amount of corresponding enzymes before sampling to cope with NO induction. *IoNOX* may be the main enzyme in *I. obliquus* in the early response to NO induction, generating reactive oxygen species, while SOD and CAT play a role in free radical scavenging in the next stage. These three enzymes dynamically coordinate to regulate the antioxidant system in response to NO signals.

### 2.5. Effect of NO Induction on Calcium Signal Transduction Gene Expression

**Figure 4:**
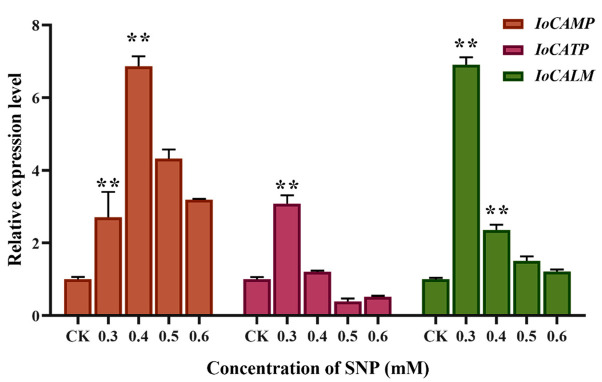
Transcriptional response of calcium ion signal transduction genes (*IoCAMP*, *IoCATP*, and *IoCALM*) after NO induction. ** represents significant differences at the *p* < 0.01 level.

Some studies have shown that abiotic stresses, such as drought and temperature, induce changes in intracellular calcium (Ca^2+^) concentration and then regulate downstream target gene expression to respond to stress signals. Figure 4 shows that the expression levels of genes involved in calcium signal transduction, calcium-binding mitochondrial carrier protein (*IoCAMP*), calcium ion binding transmembrane protein (*IoCATP*), and calmodulin (*IoCALM*) were significantly increased after NO induction. The expression level of *IoCAMP* was 6.87-fold higher than that of the control group at 0.4 mM, and the expression levels of *IoCATP* and *IoCALM* were 3.09- and 6.91-fold higher at 0.3 mM, respectively. All three calcium signal transduction genes can significantly respond to NO induction. *IoCATP* and *IoCALM* are more sensitive to NO, while *IoCAMP* shows the strongest response. These calcium signal-related proteins respond to NO treatment by synergistically regulating intracellular calcium concentrations.

## 3. Discussion

The defense mechanisms established by biological cells in response to inducers require a series of signal transductions, usually involving multiple consecutive reactions and forming regulatory networks [30,31]. Therefore, the defense process of fungal cells is usually not regulated by a single signaling pathway but involves the crosstalk of two or more signaling pathways. Current research on the regulation of terpenoid biosynthesis in *Ganoderma lucidum* by inducers has identified the collaborative involvement of signaling pathways such as jasmonic acid, antioxidant, and NO [32]. Therefore, as an important endogenous molecule, the signal transduction pathway of NO may also involve the crosstalk of multiple signal pathways. This study confirms that NO upregulates the expression levels of downstream key enzyme genes in the biosynthesis of terpenoid in *I. obliquus*, thereby increasing the accumulation of terpenoid active components such as betulin. In addition, this regulatory process may be mediated by the antioxidant and calcium ion signaling pathways.

Based on the expression levels of antioxidant signal pathway enzyme genes and activities under different NO intensities, it was found that *IoNOX* can respond most rapidly and robustly to NO signals. NADPH oxidase is expressed and produces a large amount of superoxide anions, hydrogen peroxide, and hydroxyl radicals through redox reactions for defense. Subsequently, downstream *IoSOD* and *IoCAT* also convert free radicals such as superoxide anions and hydrogen peroxide into water and oxygen through upregulated expression. These three enzymes reduce cellular oxidative damage under NO signals by dynamically regulating the antioxidant system.

Investigating the expression of calcium ion signal transduction enzyme genes under different NO intensities revealed that NO can act as a messenger for Ca^2+^ mobilization. *IoCATP* and *IoCALM* exhibit sensitivity to NO treatment. *IoCATP* activates the inward flow of Ca^2+^ from the extracellular space and causes a rapid and transient increase in cytoplasmic Ca^2+^ concentration. *IoCALM* changes its conformation by binding with calcium ions, thereby enhancing its binding ability with downstream target genes and achieving the regulation of cellular metabolic behavior. In addition, the calcium-binding mitochondrial carrier protein gene *IoCAMP* is upregulated after NO induction, regulating the concentration of calcium ions within the mitochondria. As one of the major storage pools of intracellular calcium ions, calcium concentration in mitochondria has an important impact on regulating cell death, intracellular calcium signal transduction, and energy metabolism [33].

**Figure 5:**
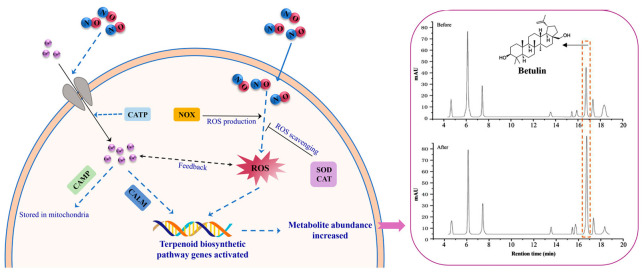
NO activates the triterpenoid biosynthetic pathway in *I. obliquus* through multilevel signaling regulation to enhance its production. Arrows indicate promotion and horizontal lines indicate inhibition. The solid line represents the single-step direct regulatory process, while the dashed line represents the multi-step regulatory process.

The upregulation of *SQS*, *SQE*, and *LUS* induced by NO is significantly positively correlated with the accumulation of betulin. Meanwhile, antioxidant and calcium signaling genes are activated, strongly suggesting the existence of a regulatory network. Triterpene biosynthesis genes require higher concentrations of NO signals to be significantly activated, while antioxidant enzymes have reached the plateau at low concentrations. This dose-dependent response might be an adaptive strategy of *I. obliquus* to cope with environmental stress. Oxidative damage is reduced by rapidly activating the antioxidant system at low concentrations of NO. High-concentration NO triggers secondary metabolism (such as triterpenoid synthesis) through specific signaling pathways (such as the synergy between Ca^2+^ and ROS) to enhance stress resistance. Many studies have shown that the mitochondrial Ca^2+^ regulatory process in eukaryotes may be closely related to the production of reactive oxygen species (ROS), and there are multiple positive and negative feedback loops between Ca^2+^ and ROS [34,35]. It is speculated that NO induction initially causes the response of the calcium ion signal transduction pathway in *I. obliquus*, then regulates the production of ROS and activates the downstream antioxidant enzyme defense system. *I. obliquus* cells further regulate the terpenoid metabolic pathway under oxidative stress signals to enhance their defensive capabilities (Figure 5). However, the upstream and downstream relationships, feedback relationships, or cascading amplification effects between the two pathways need to be further determined in subsequent studies.

In conclusion, this study investigated the inducing effect of NO on the biosynthesis of terpenoids in *I. obliquus* and its potential signal transduction mechanisms. The content of triterpenes, such as betulin, was significantly enhanced after the addition of SNP. The expression of multiple key enzyme genes in the triterpenoid biosynthetic pathway was upregulated after NO induction, confirming that NO promotes the synthesis and increases the content of triterpenes by activating the transcription of downstream enzyme genes in its biosynthetic pathway. The response of triterpenoid biosynthetic enzyme genes to NO may be achieved through signal transduction involving Ca^2+^ and ROS pathways. The specific process involves the participation of the calcium-ion-concentration-regulation-related proteins *IoCAMP*, *IoCATP*, and *IoCALM* in the calcium signaling process, as well as the dynamic cooperation of the oxidative-stress-related proteins *IoNOX*, *IoSOD*, and *IoCAT*. This study provides a theoretical basis for clarifying the molecular mechanism of the NO-mediated regulation of triterpenoid biosynthesis and provides the foundation for the development of strategies to increase the triterpenoids yield in *I. obliquus*.

## 4. Materials and Methods

### 4.1. Strain and Culture

The *I. obliquus* was preserved in the School of Biological Engineering of Dalian Polytechnic University. Solid potato dextrose agar medium was used to activate the strain. The mycelium covered the entire plate after culturing at 28 °C for about 10 days. Then, 20 pieces of mycelium (5 cm^2^) were inoculated into 10 mL of liquid medium for fermentation culturing. The liquid medium was composed of 2% potato dextrose broth, 0.4% peptone, 0.15% KH_2_PO_4_, and 0.1% MgSO_4_. The culture was maintained at 28 °C. The growth curve was plotted by measuring the biomass at different times, and the optimal sampling time was determined. To ensure the accuracy of the experimental results, the inoculated mycelium for each was from the same batch.

### 4.2. Treatment of I. obliquus Liquid Fermentation with Nitric Oxide Donor

The nitric oxide donor SNP (Shanghai Macklin Biochemical Technology Co., Ltd., Shanghai, China) was dissolved in sterile water to prepare a stock solution of 100 mmol/L, which was then filtered through a 0.22 µm membrane. Different volumes of the stock solution were added to the liquid medium to achieve final concentrations of 0.3, 0.4, 0.5, 0.6, and 0.7 mmol/L. The release of NO from sodium nitroprusside is a gradual process, and its release rate is related to environmental factors such as temperature, pH, and light. Although SNP is in a dark state when added to the medium for *I. obliquus* culturing, it is conducive to improving the stability of SNP. However, it will still be completely decomposed within 24 h. Therefore, SNPS are supplemented regularly every 24 h to ensure the concentration of NO. The medium without SNP was used as the control. The mycelium was collected at different times for subsequent analysis.

### 4.3. Determination of Mycelial Biomass and Microsphere Diameter

The mycelium spheres were collected after fermentation for 5 days under different concentrations of SNP, washed with sterile water until the filtrate was clear, and dried at 50 °C to a constant weight. The mycelial biomass (dry weight) was determined by weighing. In addition, the fermentation broth was transferred to a beaker, and 20 microspheres were randomly selected and tightly arranged in a row to measure the total length, and the average diameter of the spheres was calculated.

### 4.4. Determination of Triterpenoids Content

Considering the lag in metabolite accumulation, the mycelium spheres of *I. obliquus* fermented under different concentrations of SNP induction were collected after 7 days of cultivation. An amount of 0.1 g of ground mycelial powder was weighed, and the triterpenoids were extracted with 1 mL of 90% (*v*/*v*) ethanol aqueous solution for 2 h under ultrasound. The supernatant was collected after centrifugation, and the extraction was repeated three times. The supernatants were combined. The solvent was removed by rotary evaporation, and then the sample was brought to a volume of 1 mL with chromatographic methanol. After filtering through a membrane, the sample was used for the high-performance liquid chromatography (HPLC) detection of triterpenoids.

The abundance of triterpenoid components was determined using HPLC (Waters Alliance 2695, Milford, MA, USA) equipped with an Agilent ZORBOX Eclipse Plus C18 (5 µm, 4.6 × 250 mm) column. The mobile phase was methanol (A) and water (B), with a gradient of 0 min, 20% A; 0–5 min, 40% A; 5–10 min, 40–95% A, 10–12 min, 95–100% A, 12–20 min, 100% A. The flow rate was 1.0 mL/min, and the injection volume was 10 μL. The detection wavelength was 210 nm. The column temperature was maintained at 25 °C. For the triterpenoid compound with the most significant difference in peak area at a retention time of around 16.7 min under NO induction, a variety of standards were used for retention time comparison. Three identified compounds in *I. obliquus* were selected as standards, betulin (Cas: 473-98-3), betulinic acid (Cas: 472-15-1), and inotodiol (Cas: 35963-37-2), for retention time comparison. All standards were obtained from Shanghai Yuanye Bio-Technology Co., Ltd. (Shanghai, China) It was finally identified that the compound separated at this retention time was betulin. A standard curve was plotted by the response value of different concentrations (1, 0.5, 0.25, and 0.125 mg/mL) of betulin standard solution (R^2^ > 0.99), the betulin in the *I. obliquus* was quantitatively determined according to the peak area (Figure S4).

### 4.5. Antioxidant Enzyme Activity Assay

An amount of 0.2 g of mycelial powder was mixed with liquid nitrogen, then 2 mL of pre-cooled phosphate buffer (pH 7.8) was added and fully ground into homogenize. The supernatant was taken after centrifuging at 4 °C, and the volume was adjusted to 5 mL with phosphate buffer to obtain the crude enzyme solution of superoxide dismutase (SOD). The absorbance at 560 nm was measured using the nitro blue tetrazolium (NBT) method to calculate the activity of SOD. The control group was not added with samples but only with buffer solution, while the sample group was added with samples.

Activity of SOD (U/g) = (ΔA_control_ − ΔA_sample_) × 5/2.15 × 10^4^ × 1 × 0.2

An amount of 0.2 g of mycelial powder was mixed with liquid nitrogen, then 2 mL of pre-cooled phosphate buffer (pH 6.0) was added and fully ground into homogeneity. The supernatant was taken after centrifuging at 4 °C, and the volume was adjusted to 5 mL with buffer to obtain the crude enzyme solution of peroxidase (POD). The activity of POD was determined by measuring the absorbance at 470 nm using the guaiacol method. The control group was not added with samples but only with buffer solution, while the sample group was added with samples.

Activity of POD (U/g) = (ΔA_control_ − ΔA_sample_) × 5/2.66 × 10^4^ × 1 × 0.2

An amount of 0.1 g of mycelial powder was weighed, and the crude enzyme solution of NADH Oxidase was extracted using an NADH Oxidase (NOX) Activity Assay Kit (Solarbio, Beijing, China), and the absorbance was measured at 600 nm. The activity of NOX was calculated based on the reduction rate of 2,6-dichlorophenolindophenol. The control group was not added with samples but only with buffer solution, while the sample grCoup was added with samples.

Activity of NOX (U/g) = (ΔA_control_ − ΔA_sample_) × V_total_/2.1 × 10^4^ × 1 × 0.1

### 4.6. Real-Time Quantitative PCR Analysis

After fermentation with different concentrations of SNP for 5 days, the mycelium spheres of *I. obliquus* were collected and ground in liquid nitrogen. Total RNA was extracted using a StarPure RNA Kit (GenStar, Beijing, China) according to the manufacturer’s protocol. The quality of the total RNA was determined by an absorbance ratio at 260 nm/280 nm and agarose gel electrophoresis (Figure S5). After obtaining pure RNA without impurities, reverse transcription was performed. Real-time fluorescence quantitative PCR analysis (qRT-PCR) was carried out using a LightCycler^®^ 480 SYBR (Roche, Basel, Switzerland) with cDNA obtained from different experimental groups as templates. Primers were designed using Primer 5 software for genes related to triterpenoid biosynthesis, oxidative defense, and calcium ion signaling (Table S1). The primer sequences were shown in (Table S2). The PCR reaction system was 10 μL, including 1 μL diluted cDNA, 5 μL PS-qMix, 0.2 μL forward primer, 0.2 μL reverse primer, and 3.66 μL ultrapure water. The amplification conditions were 5 min at 95 °C, 40 cycles of 95 °C for 20 s, 50 °C for 15 s, and 72 °C for 15 s, followed by 72 °C for 10 min. The relative mRNA levels were normalized to the internal control gene (β-actin) and the corresponding values with the control group were compared.

### 4.7. Statistical Analysis

All experiments were set up with three biological replicates. A one-way ANOVA was performed using GraphPad Prism 9.0 software to calculate the statistical significance of the differences. The difference was considered to be significant when *p* < 0.05 and expressed as “*”. An extremely significant difference was expressed with “**” when *p* < 0.01.

## Data Availability

The original data of this present study are available from the corresponding authors.

## Supplementary Materials

The following supporting information can be downloaded at: https://www.mdpi.com/article/10.3390/ijms26104561/s1.
