# Supplementary material for: NO Activates the Triterpenoid Biosynthetic Pathway in Inonotus obliquus Through Multilevel Signaling Regulation to Enhance Its Production
Source: Int J Mol Sci. 2025 May 9;26(10):4561. doi: 10.3390/ijms26104561 (PMC12110904; doi:10.3390/ijms26104561)
Supplement: Supplementary file 1 [file ijms-26-04561-s001.zip › ijms-3574184-supplementary.pdf]

Supplementary Materials Data

NO Activates the Triterpenoid Biosynthetic Pathway in *Inonotus obliquus* Through Multilevel Signaling Regulation to Enhance Its Production

Ping Kou *, Yu-Chi Zhang, He Wang, Li-Li Mo, Jun-Jiao Gu and Fang Yu

School of Biological Engineering, Dalian Polytechnic University, Dalian 116034, China
* Correspondence: kouping@dlpu.edu.cn

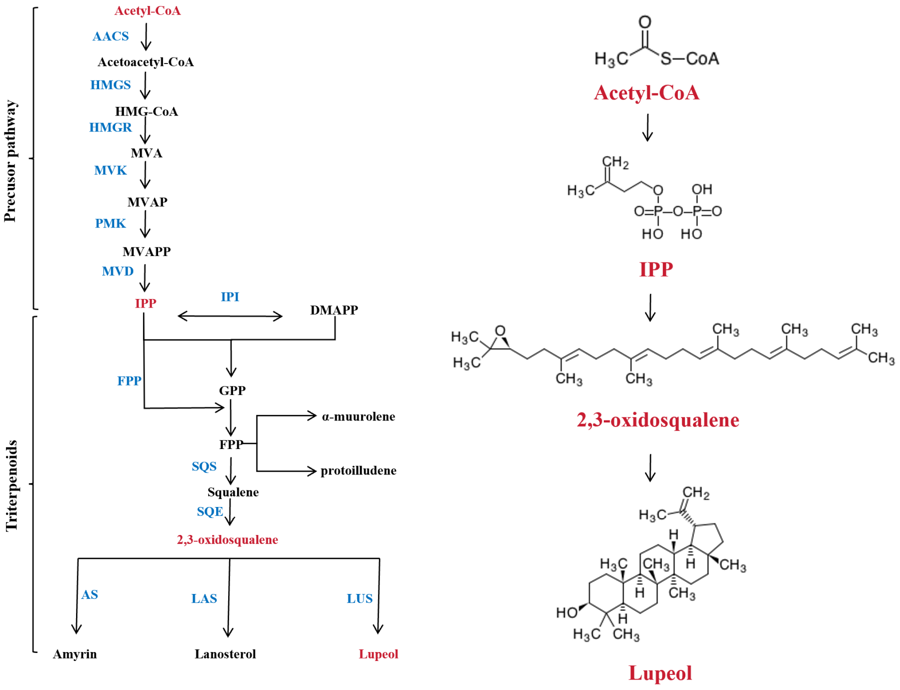

Figure S1. The biosynthetic pathway of terpenoids.

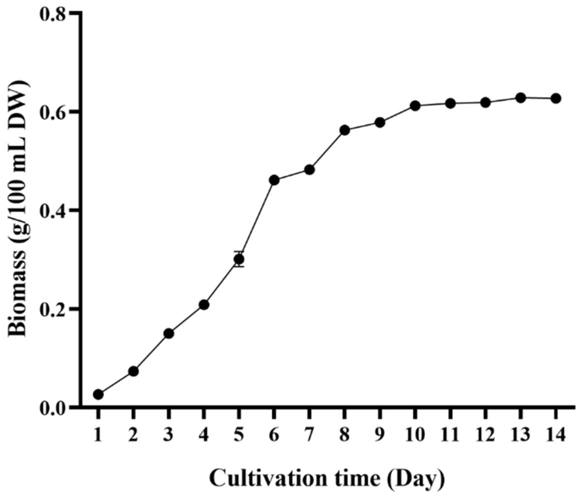

Figure S2. Growth profile of *Inonotus obliquus* during liquid fermentation.

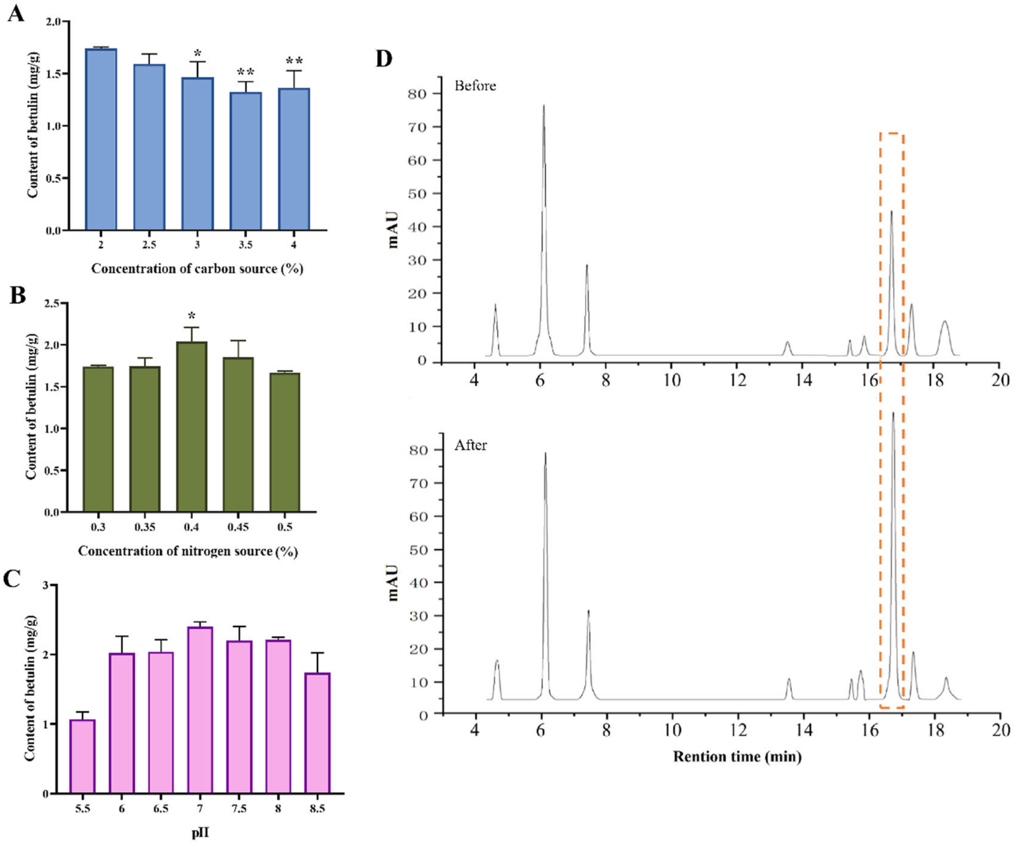

Figure S3. Optimization of carbon source, nitrogen source and pH during liquid fermentation of *I. obliquus* (**A**–**C**) and HPLC detection of betulin before and after optimization (**D**).

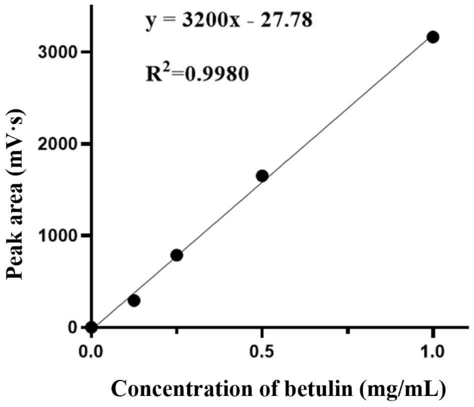

Figure S4. Standard curve of betulin.

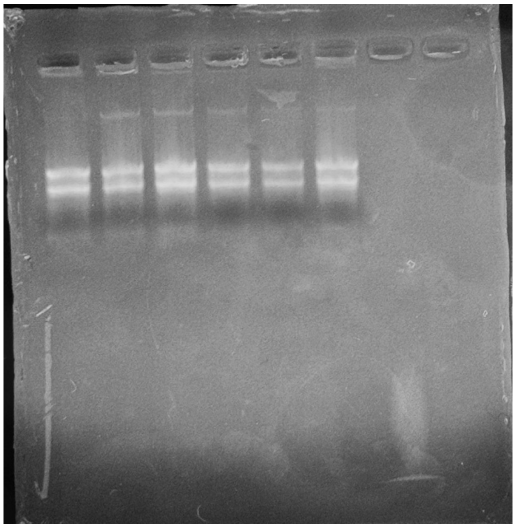

Figure S5. Uncropped gel electrophoresis of total RNA in control group and experimental groups.

Table S1. GenBank accession for triterpenoid biosynthetic genes.

Genes	GenBank Accession
IoHMGR	JN580310.1
IoMVD	MK82558
IoFPP	MK825560.1
IoSQS	JN580311.1
IoSQE	MK825566.1
IoLUS	OP810572.1

Table S2. Primers used in this study.

Primers	Sequences (5′-3′)
actin-F	CCACGAGACAACATACAACT
actin-R	CCACGAGACAACATACAACT
IoHMGR-F	ACATCCTCACGGCGATCTTCCT
IoHMGR-R	GCGTCATCATTCGTTGGCTCCA
IoMVD-F	CAGTTTCTGGAGGTGGTGCC
IoMVD-R	AAGGGGATAAGGGATGTGGA
IoFPP-F	CATCTCTGCCTTTGCCCTGT
IoFPP-R	TCCAGGCAACTCGTGGCTTA
IoSQS-F	AGCAGGTGTGACGGCAAACG
IoSQS-R	GACGAIGGCGAACGCAAGGA
IoSQE-F	GCAAGGAGGTGGAGACAA
IoSQE-R	GACCCAGAGGCATGGAAA
IoLUS-F	ATTGGGGGATTGCTACACCTACG
IoLUS-R	GTCCACACCTTGCTTTGGCTTGA
IoNOX-F	ATAACCGTTGACCGGCCATT
IoNOX-R	AAGATGAGTTTCCGCGGTGT
IoSOD-F	GACGAAGGTGGAGAGTGCAA
IoSOD-R	TGCAGAGTATTCGGGTTCCG
IoCAT-F	CCGGGATCAAACCATGTGGA
IoCAT-R	GGTGGCTGGCAGGTAAATCT
IoCAMP-F	CTTCCTACGTTGACCACGCT
IoCAMP-R	TCGCGGAGAATTCGCTCTAC
IoCATP-F	GGGCACAAGGTGATCGAGAA
IoCATP-R	CGTCCAACACAGTTCCGAGA
IoCALM-F	GGTGTTCGATAAGGACGGCA
IoCALM-R	AATCATCTCGTCGACCTCGC
